# Computational pharmaceutical materials science

**DOI:** 10.1186/1758-2946-6-S1-O21

**Published:** 2014-03-11

**Authors:** Jacco van de Streek

**Affiliations:** 1 Department of Pharmacy, University of Copenhagen, Copenhagen, 2100, Denmark

## 

Pharmaceutical materials science addresses the physical solid-state properties of pharmaceutical products, such as solubility and stability of crystal structures. Especially the different properties of different polymorphs, salts and hydrates of an Active Pharmaceutical Ingredient (API) are of interest, although more recently the amorphous state has also received considerable attention.

Calculations, such as dispersion-corrected Density Functional Theory [1] (DFT-D), can help to determine crystal structures from challenging experimental data (e.g., structure determination from X-ray powder diffraction data [2]) and can help to identify the most stable form of an API (crystal structure prediction [3]). Molecular Dynamics (MD) can be used to resolve disorder and phase transitions in crystal structures, and more recently MD is being used to investigate the amorphous state.

This presentation will give several examples of the projects that are currently underway in our group.
